# Intravitreal Administration of rhNGF Enhances Regenerative Processes in a Zebrafish Model of Retinal Degeneration

**DOI:** 10.3389/fphar.2022.822359

**Published:** 2022-03-07

**Authors:** Pasquale Cocchiaro, Vincenzo Di Donato, Davide Rubbini, Rodolfo Mastropasqua, Marcello Allegretti, Flavio Mantelli, Andrea Aramini, Laura Brandolini

**Affiliations:** ^1^ Dompé Farmaceutici SpA, Napoli, Italy; ^2^ ZeClinics SL, IGTP (Germans Trias I Pujol Research Institute), Barcelona, Spain; ^3^ Institute of Ophthalmology, University of Modena and Reggio Emilia, Modena, Italy

**Keywords:** nerve growth factor (NGF), age-related macular degeneration (AMD), retinitis pigmentosa (RP), zebrafish, retinal regeneration, Müller glia cells, neuroprotection, translational research

## Abstract

Nerve growth factor (NGF) is the best characterized neurotrophin, and it is known to play an important role in ocular homeostasis. Here, we demonstrated the expression of NGF receptors in adult zebrafish retina and optimized a light-induced retina degeneration (LID) zebrafish model that mimics human cone-rod disorders, demonstrating that intravitreal (IV) administration of rhNGF can boost zebrafish retinal regeneration in this model. Adult zebrafish retinae exposed to 60 h of light irradiation (60 h LID) displayed evident reduction of outer nuclear layer (ONL) thickness and cell number with presence of apoptotic cells. Retinal histologic evaluation at different time points showed that IV therapeutic injection of rhNGF resulted in an increase of ONL thickness and cell number at late time points after damage (14 and 21 days post injury), ultimately accelerating retinal tissue recovery by driving retinal cell proliferation. At a molecular level, rhNGF activated the ERK1/2 pathway and enhanced the regenerative potential of Müller glia *gfap-* and *vim-*expressing cells by stimulating at early time points the expression of the photoreceptor regeneration factor Drgal1-L2. Our results demonstrate the highly conserved nature of NGF canonical pathway in zebrafish and thus support the use of zebrafish models for testing new compounds with potential retinal regenerative properties. Moreover, the pro-regenerative effects of IV-injected NGF that we observed pave the way to further studies aimed at evaluating its effects also in mammals, in order to expedite the development of novel rhNGF-based therapeutic approaches for ophthalmological disorders.

## Introduction

Inherited, acquired, or iatrogenic, the retinal conditions are complex and multi-factorial diseases characterized by progressive bilateral degeneration of the rod and cone photoreceptors (Narayan et al., 2016; Al-Zamil and Yassin 2017). Photoreceptor degeneration ultimately leads to either partial or total visual impairment, thus severely compromising the quality of life of affected individuals (Teutsch et al., 2016; Welp et al., 2016). Over the years, several potential therapeutic approaches have been developed with the aim of delaying the progression of these diseases (Connors et al., 2021), but currently, a therapy that is able to promote the reversion of the phenotype remains an unmet medical need.

Photoreceptor apoptotic death is the final step of several retinal disorders, and the deregulation of the nerve growth factor (NGF) pathway observed in retinal degeneration models supports the hypothesis that a profound alteration of NGF pathway balance may account for the progressive neuronal cell death and photoreceptor loss (Santos et al., 2012; Garcia et al., 2017). NGF is the best characterized member of the neurotrophin family and has been widely described as a critical neuronal survival factor (Colafrancesco et al., 2011; L.; Mesentier-Louro et al., 2017; L. A.; Mesentier-Louro et al., 2018). It plays a crucial role also in regulating the homeostasis of ocular tissues (Aloe et al., 2012), where it is expressed with its receptors TrkA and p75 (Garcia et al., 2017): TrkA has high affinity and selectivity for NGF binding and can trigger different signaling pathways related to cell survival, proliferation, and differentiation, which involve the activation of ERK, PI3K, and PLC-γ (Wang et al., 2014); on the contrary, p75 has low affinity for NGF and high affinity for proNGF and is implicated in cell death mediation (Coassin et al., 2008). The role of NGF in visual function has been largely attributed to its ability to regulate phenotypic features of the neuro-retina, innervation density and plasticity (Sacchetti et al., 2017), cell body size, axonal terminal sprouting, and dendritic growth (Rocco et al., 2018). Notably, increasing lines of evidence have shown that NGF can be crucial for the treatment of blinding diseases, such as retinal degenerations, and several studies have already described the protective effect of NGF administration in experimental models of retinitis pigmentosa (RP) (Rocco et al., 2015) and age-related macular degeneration (AMD) (Lambiase et al., 2009).

In eye research, the availability of appropriate animal models has been extremely valuable to investigate pathological molecular mechanisms and to test new therapeutic interventions (Shah et al., 2019). Among the various species, zebrafish has progressively become a powerful model system especially for studying complex diseases, as the conservation between organization and function in human and zebrafish retinas has been a great advantage. Unlike mammals that cannot regenerate their retina after damage or degeneration, the zebrafish retina displays a robust regenerative response upon injury, and this feature can be exploited to gain insights into molecular mechanisms underlying healing of damaged tissues in eye human pathology (Wan and Goldman 2016; Richardson et al., 2017). Regeneration of the zebrafish retina (Nagashima et al., 2013; Wan and Goldman 2016) occurs *via* the ciliary marginal zone (CMZ), which is a stem cell niche that constantly adds new retinal neurons as the retina grows throughout life and *via* Müller glia (MG) cells, accounting for differentiated quiescent cells that normally help in maintaining retinal architecture and homeostasis but that can also be primed by a photic, chemical, or mechanical damage of the retina. In case of retinal injury, MG cells undergo a reprogramming event that leads to the generation of multipotent neuronal progenitors, which can then migrate and differentiate into any of the lost retinal cell type (Powell et al., 2016). The molecular circuitry driving MG reprogramming is poorly understood, but studies indicate that a remarkable variety of secreted factors contribute to retina regeneration in zebrafish and modulate MG reprogramming and proliferation in the injured retina (Faillace et al., 2002; Kassen et al., 2009; Ramachandran et al., 2011; Wan et al., 2012; Nelson et al., 2013).

Both NGF and its canonical receptors, known in the zebrafish as *ntrk1* and *ngfrb*, are conserved and widely expressed in zebrafish neurons, suggesting a conserved pathway activation *via* binding of neuronal cell surface receptors (Götz and Schartl 1994; Martin et al., 1995; Nittoli et al., 2018; Cacialli et al., 2019; Hahn et al., 2020).

Here, we sought to assess the potential regenerative effect of intravitreal (IV) administration of rhNGF using a retinal degeneration paradigm in adult zebrafish based on constant light irradiation.

Our results show that NGF receptors are expressed in adult zebrafish retina and that administration of rhNGF can boost zebrafish retinal regeneration upon injury.

## Results

### Expression Pattern of *ngfrb* and *ntrk1* in Zebrafish Adult Retinae

**FIGURE 1:**
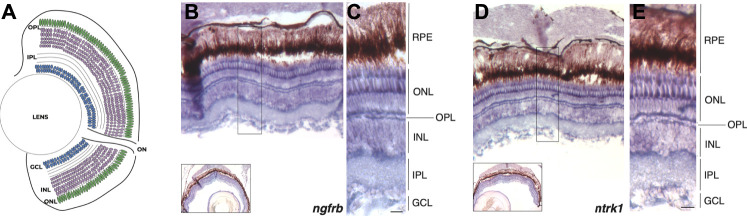
*ngfrb* and *ntrk1* mRNA expression pattern in adult zebrafish eye. **(A)** Schematic representation of the zebrafish retina with nuclear and plexiform layers highlighted. **(B)** *In situ* hybridization (ISH) for *ngfrb* expression in cross sections of adult zebrafish retina (signal in blue/violet). **(C)** High magnification showing the expression of *ngfrb* gene in the different retinal layers and cell types. **(D)** ISH for *ntrk1* expression in adult zebrafish retina (signal in blue/violet). **(E)** High magnification showing *ntrk1* transcript localization in the different retinal layers and cell types. RPE: retinal pigmented epithelium. ONL: outer nuclear layer. OPL: outer plexiform layer. INL: inner nuclear layer. IPL: inner plexiform layer. GCL: ganglion cell layer. Scale bar: 50 μm.

Although a recent study confirmed the expression of *ngfrb* (ortologue of mammalian p75) and *ntrk1* (ortologue of mammalian TrkA) genes in sensory neurons during zebrafish early development (Hahn et al., 2020), the expression of the two NGF receptors in adult zebrafish retina has not been reported yet. Thus, in order to evaluate the potential regenerative effect of rhNGF administration in adult zebrafish retinal degeneration model, we first assessed the expression of NGF receptors in adult zebrafish retina. With this aim, we performed *in situ* hybridization (ISH) on retinal sections for detection of *ngfrb* and *ntrk1* in adult zebrafish eyes. Transcript localization of *ngfrb* and *ntrk1* revealed a mild widespread expression in the adult zebrafish retina and showed that both NGF receptors are expressed predominantly in the photoreceptor cells of the outer nuclear cell layer (ONL) (Figure 1). These data demonstrate that NGF receptors are present in the adult zebrafish retina, thus suggesting that the tissue is potentially responsive to endogenous or exogenous NGF.

### Optimization of a Zebrafish-Based Retinal Degeneration Paradigm for Ophthalmological Studies

**FIGURE 2:**
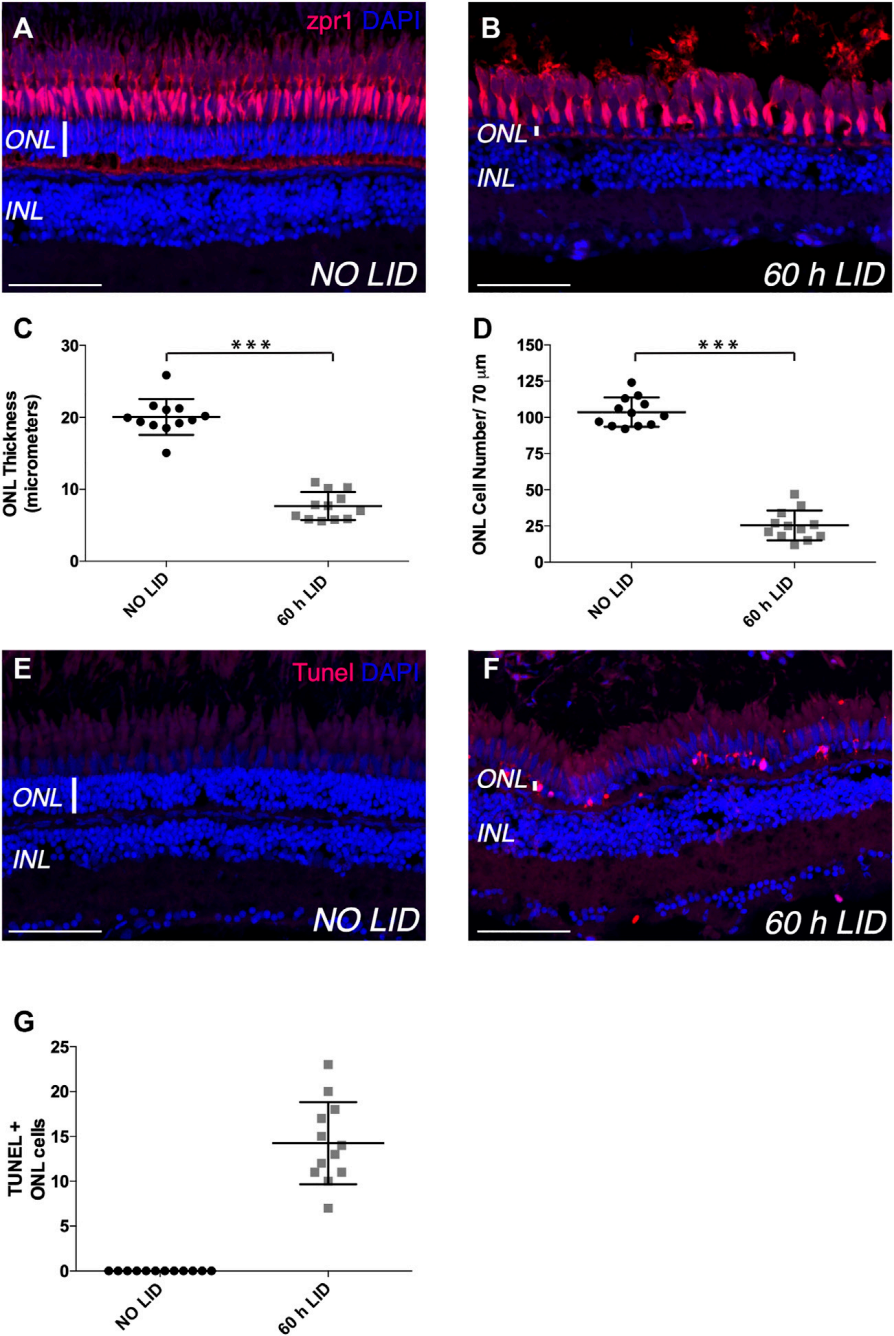
Light-induced retinal degeneration zebrafish model. **(A,B)** Representative images of retinal cryosections of adult zebrafish eyes immunostained with Zpr1 antibody (red), which labels photoreceptor cells, and stained with DAPI (blue), which labels nuclei. **(A)** Retinal cryosection of adult zebrafish eye not exposed to constant light (NO LID). **(B)** Retinal cryosection of adult zebrafish eye exposed to 60 h of constant light (light-induced degeneration—LID). **(C)** Quantification of ONL thickness (number of retinae analyzed = 12). **(D)** Quantification of ONL cell number (number of retinae analyzed = 12, total number of cells: 305 for injured and 1,243 for control retinae). **(E,F) **Representative images of retinal cryosections of adult zebrafish eyes stained with TUNEL (red), which labels apoptotic cells, and with DAPI (blue), which labels nuclei. **(E)** Retinal cryosection of adult zebrafish eye not damaged by light exposure (NO LID). **(F)** Retinal cryosection of adult zebrafish eye after 60 h of LID. **(G)** Quantification of TUNEL-positive cells shown as mean number per retina (number of retinae analyzed = 12). Data are shown as means ± SEM. ***p < 0.001 (Mann–Whitney test). Scale bar: 50 μm.

Since loss of photoreceptors is a hallmark of human retinal conditions such as AMD and RP (Marc et al., 2003; Kalloniatis and Fletcher 2004), we sought to assess whether IV administration of rhNGF might have a regenerative effect on photoreceptor cell population upon damage. With this aim, we first optimized a zebrafish model of retinal degeneration induced by constant light irradiation. Injury models based on photo-induced retinal degeneration have been developed in several species (Donovan et al., 2001; Saito et al., 2016; Sudharsan et al., 2017) and showed that constant light exposure induces loss of rod and cone photoreceptors and, macroscopically, thinning of the retinal outer nuclear layer (ONL), where these photoreceptors are located. Since fish have long-life ability to heal the retinal tissue, we first had to establish the appropriate time of light exposure needed to induce degeneration of the retinal tissue. Thus, after a period of dark acclimatization of 14 days, adult zebrafish were exposed to constant light (18,000–20,000 lumens) and eyes were enucleated at time 0 (not exposed to light) and then at different time points after light irradiation onset. Immunohistochemistry with Zpr1 antibody on enucleated eyes was performed to label red/green double cone photoreceptors (Matsuoka et al., 2013), used as reference cell population of the ONL, and thickness and cell number of ONL were established as phenotypic readouts to assess the severity of the injury. Initially, we exposed adult zebrafish to constant bright light for 48 h, as it was previously described as a stimulus able to induce a mild to drastic reduction of photoreceptors (Thomas et al., 2012; Saito et al., 2016). However, in our hands, 48 h of light-induced degeneration (48 h LID) only slightly reduced ONL thickness and cell number compared to non-injured retinae (NO LID) (Supplementary Figure S1). In order to induce a more consistent damage to photoreceptors, we increased the light treatment to 60 h, and indeed, at this time point, the integrity of double cone photoreceptors, in terms of density, was severely compromised compared to non-injured retinae (NO LID) (Figure 2A,B). Moreover, photoreceptor layer damage was confirmed by evident reduction of ONL thickness and cell number in LID retinae (Figure 2C,D). Injured retinal tissue displayed on average a significantly thinner ONL (7.671 ± 0.5606 SE μm) characterized by a significantly lower number of cells (25.42 ± 2.953 SEM) compared to control retinae thickness (20.05 ± 0.7203 SE μm) and cell number (103.6 ± 2.904 SEM). To further assess the injury effect on cell viability, we performed terminal deoxynucleotidyl transferase-mediated dUTP nick-end labeling (TUNEL) assay for the detection of apoptotic photoreceptors. While no cell death could be observed in the ONL of control retinae (NO LID), several apoptotic cells were detected upon 60 h of light treatment (Figure 2E,G) in retinal tissues analyzed.

Based on these results, we determined that 60 h was the most appropriate light exposure time needed to induce degeneration of adult zebrafish retinal tissue in our model.

### rhNGF Enhances Retinal Regeneration After Light-Induced Damage

Having defined the appropriate retina degeneration conditions (60 h LID), we used this experimental model to test the potential effect of rhNGF on zebrafish retinal recovery upon injury. To this end, we administered 5 μg/eye of rhNGF through eye incision and posterior IV injection in adult zebrafish immediately after 60 h of light exposure. Thereafter, groups of fish were sacrificed at five different time points [0 days post injury (dpi), 7 dpi, 14 dpi, 21 dpi, and 28 dpi] and eyes were removed. Incised and non-rhNGF-injected contralateral eyes were enucleated at the same time points and used as control, as no differences—in terms of ONL thickness and ONL cell number—were observed in a preliminary study in which we compared 60 LID non-injected (sham) and 60 LID saline-injected animals (data not shown).

**FIGURE 3:**
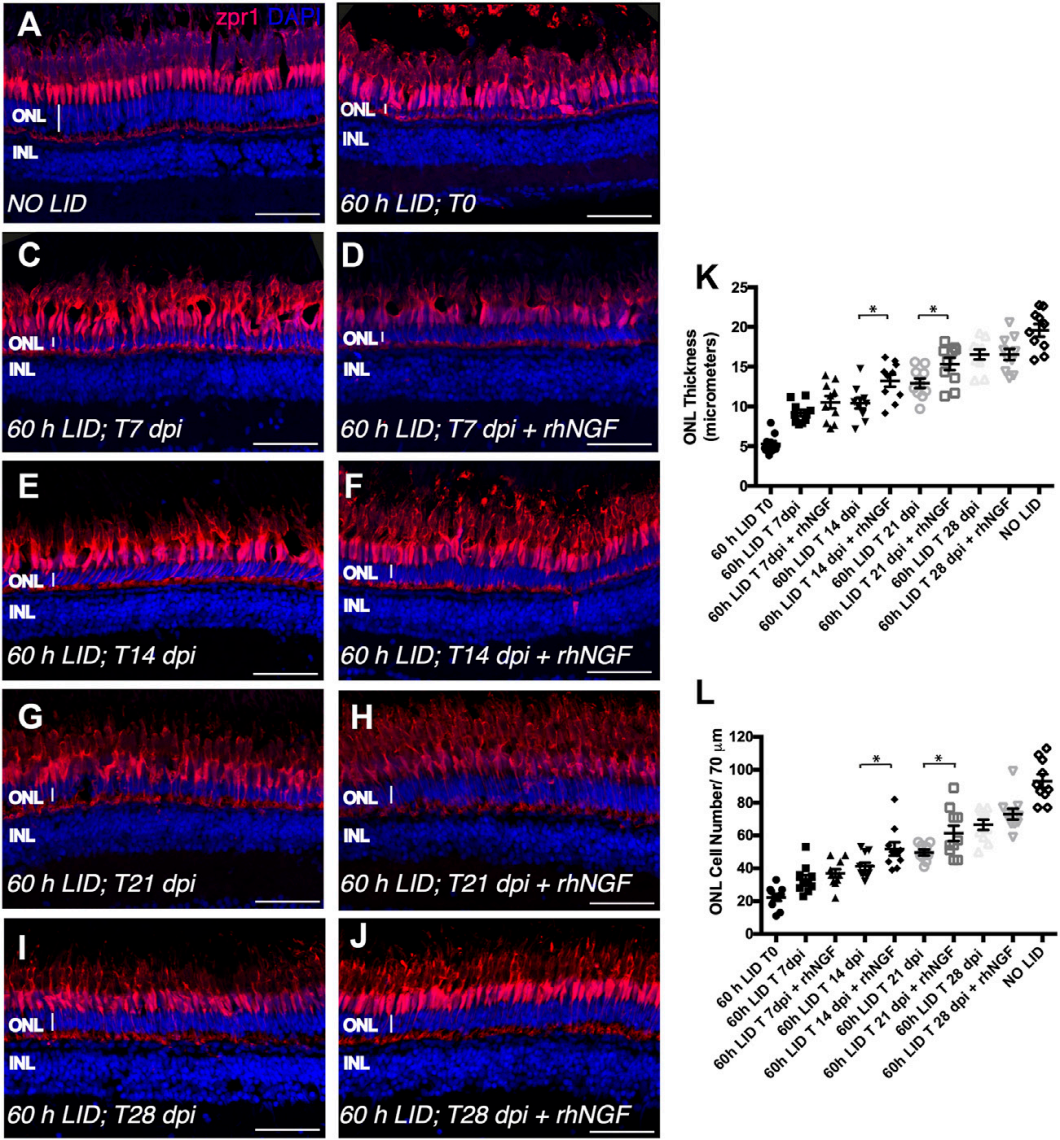
Analysis of rhNGF-induced retinal tissue recovery upon light-induced degeneration (LID). (A–J) Representative images of retinal cryosection of adult zebrafish eyes immunostained for Zpr1 antibody (red) and stained with DAPI (blue). **(A)** Retinal cryosection of an adult zebrafish eye not exposed to light. **(B)** Retinal cryosection of an adult zebrafish eye exposed to 60 h of LID at T = 0 (*N* animals:10; *N* total cells: 223). **(C)** Cryosection of an untreated adult zebrafish retina at 7 dpi (*N* animals:10; *N* total cells: 332). **(D)** Retinal cryosection of rhNGF-injected adult zebrafish eye at 7 dpi (*N* animals:10; *N* total cells: 369). **(E)** Cryosection of an untreated adult zebrafish retina at 14 dpi (*N* animals:10; *N* total cells: 412). **(F)** Retinal cryosection of rhNGF-injected adult zebrafish eye at 14 dpi (*N* animals:10; *N* total cells: 518). **(G)** Cryosection of an untreated adult zebrafish retina at 21 dpi (*N* animals:10; *N* total cells: 498). **(H)** Retinal cryosection of rhNGF-injected adult zebrafish eye at 21 dpi (*N* animals:10; *N* total cells: 614). **(I)** Cryosection of an untreated adult zebrafish retina at 28 dpi (*N* animals:10; *N* total cells: 665).**(J)** Retinal cryosection of rhNGF-injected adult zebrafish eye at 28 dpi (*N* animals:10; *N* total cells: 731). **(K)** Quantification of ONL thickness in the different conditions. Data are shown as means ± SEM: *p < 0.05 (one-way ANOVA test followed by a Sidak’s multiple comparison test); 60 h LID T 14 dpi vs. 60 h LID T 14 dpi + rhNGF: Adjusted *p*-value: 0.0108; 60 h LID T 21 dpi vs. 60 h LID T 21 dpi + rhNGF: Adjusted *p*-value: 0.0394. **(L)** Quantification of ONL cell number in the different conditions. Data are shown as means ± SEM: *p < 0.05 (one-way ANOVA test followed by a Sidak’s multiple comparison test); 60 h LID T 14 dpi vs. 60 h LID T 14 dpi + rhNGF: Adjusted *p*-value: 0.0424; 60 h LID T 21 dpi vs. 60 h LID T 21 dpi + rhNGF: Adjusted *p*-value: 0.0240. DPI: days post injury. Scale bar: 50 μm.

As previously described, the readouts of the experiment were the ONL thickness and cell number per retinal length (Figure 3). As expected, at 0 dpi, analyzed retinal tissue displayed a reduced ONL (5.295 ± 0.3808 SEM μm) that was characterized by a low number of cells (22.30 ± 2.129 SEM) (Figure 3B). At 7 dpi, a comparable recovery in both readouts was observed in both rhNGF-injected and untreated retinae (Figure 3C,D). Notably, at 14 dpi, the recovery in rhNGF-injected retinae was significantly higher compared to untreated retinae and was demonstrated by an amelioration of ONL thickness and cell number (Figure 3E,F,K,L). A similar result was observed at 21 dpi, when rhNGF-treated retinal tissue displayed an improved recovery rate in both parameters compared to the untreated retinae (Figure 3G,H,K,L). At 28 dpi, treated and untreated retinae showed similar values of ONL thickness and cell number (Figure 3I,J).

**FIGURE 4:**
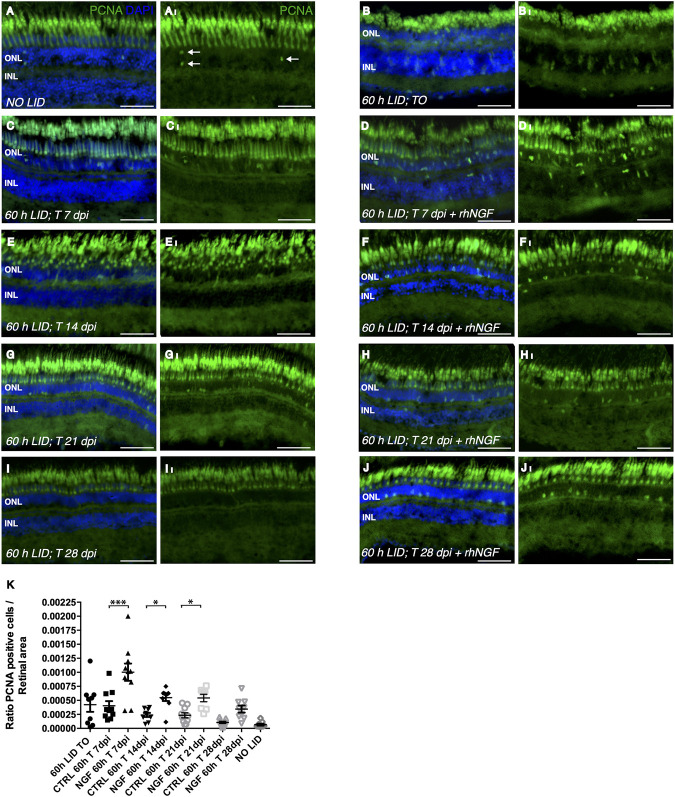
Analysis of rhNGF-induced cell proliferation upon light-induced degeneration (LID). (A–J) Representative images of retinal cryosection of adult zebrafish eyes immunostained with anti-PCNA antibody, which labels proliferating cells (green), and stained with DAPI (blue). (A_I_–J_I_) Images extrapolated from A–J displaying exclusively the PCNA signal. **(A,A)_I_** Retinal cryosection of an adult zebrafish eye not exposed to light (*N* retinae: 9). **(B,B_**I**_)** Retinal cryosection of adult zebrafish eye exposed to 60 h of LID (*N* retinae: 9). **(C,C_**I**_)** Cryosection of untreated adult zebrafish retina at 7 dpi (*N* retinae: 10). **(D,D_**I**_**) Retinal cryosection of rhNGF-injected adult zebrafish eye at 7 dpi (*N* retinae: 10). **(E,E_**I**_)**. Cryosection of untreated adult zebrafish retina at 14 dpi (*N* retinae: 9). **(F,F_I_)** Retinal cryosection of rhNGF-injected adult zebrafish eye at 14 dpi (*N* retinae: 9). **(G,G_**I**_)** Cryosection of untreated adult zebrafish retina at 21 dpi (*N* retinae: 10).**(H,H_**I**_) **Retinal cryosection of rhNGF-injected adult zebrafish eye at 21 dpi (*N* retinae: 8). **(I,I**_**I**_ **)** Cryosection of untreated adult zebrafish retina at 28 dpi (*N* retinae: 9). **(J,J_**I**_)** Retinal cryosection of untreated rhNGF-injected adult zebrafish eye at 28 dpi (*N* retinae: 9).(K) Quantification of proliferating cells. Ratio of PCNA-positive cells over the analyzed area is shown. Data are shown as means ± SEM (*n* = 10). ***p < 0.001; **p < 0.01; *p < 0.05 (one-way ANOVA test followed by a Sidak’s multiple comparison test); 0 h LID T 7 dpi vs. 60 h LID T 7 dpi + rhNGF: Adjusted *p*-value:< 0.001; 60 h LID T 14 dpi vs. 60 h LID T 14 dpi + rhNGF: Adjusted *p*-value: 0.0379; 60 h LID T 21 dpi vs. 60 h LID T 21 dpi + rhNGF: Adjusted *p*-value: 0.0412. DPI: days post injury. Scale bar: 50 μm.

To further investigate whether the improved condition of the photoceptor cell layer observed in rhNGF-treated retinae compared to untreated tissue was determined by an increase in cell proliferation or a reduction in apoptotic processes, we analyzed cell proliferation and apoptosis on retinae of the same groups of animals sacrificed for the ONL evaluation at all five time points. Cell proliferation was evaluated by immunohistochemical analysis of proliferation cell nuclear antigen (PCNA)-positive cells. Although, after 60 h of light irradiation, some degree of cell proliferation was visible also in untreated retinae compared to retinae of fish not exposed to light, a significantly higher number of dividing cells per tissue area was detected in retinae from rhNGF-injected eyes compared to untreated ones at all the analyzed time points, with the exception of 28 days, the time point corresponding to a *plateau* of tissue recovery, as also described in previous reports (Figure 4) (Thummel et al., 2008a; Thummel et al., 2008b; Thomas et al., 2016). In contrast, photoreceptor apoptosis detected by TUNEL staining did not vary between treated and non-treated groups over the 28 days of analysis (Supplementary Figure S2).

Overall, these results suggest that IV injection of rhNGF induces retinal tissue recovery by driving cell proliferation upon injury in our zebrafish retinal degeneration model, whereas it has no effect on photoreceptor cell death.

### rhNGF-Mediated Pathway Activation and Gene Expression After Light-Induced Retinal Degeneration

**FIGURE 5:**
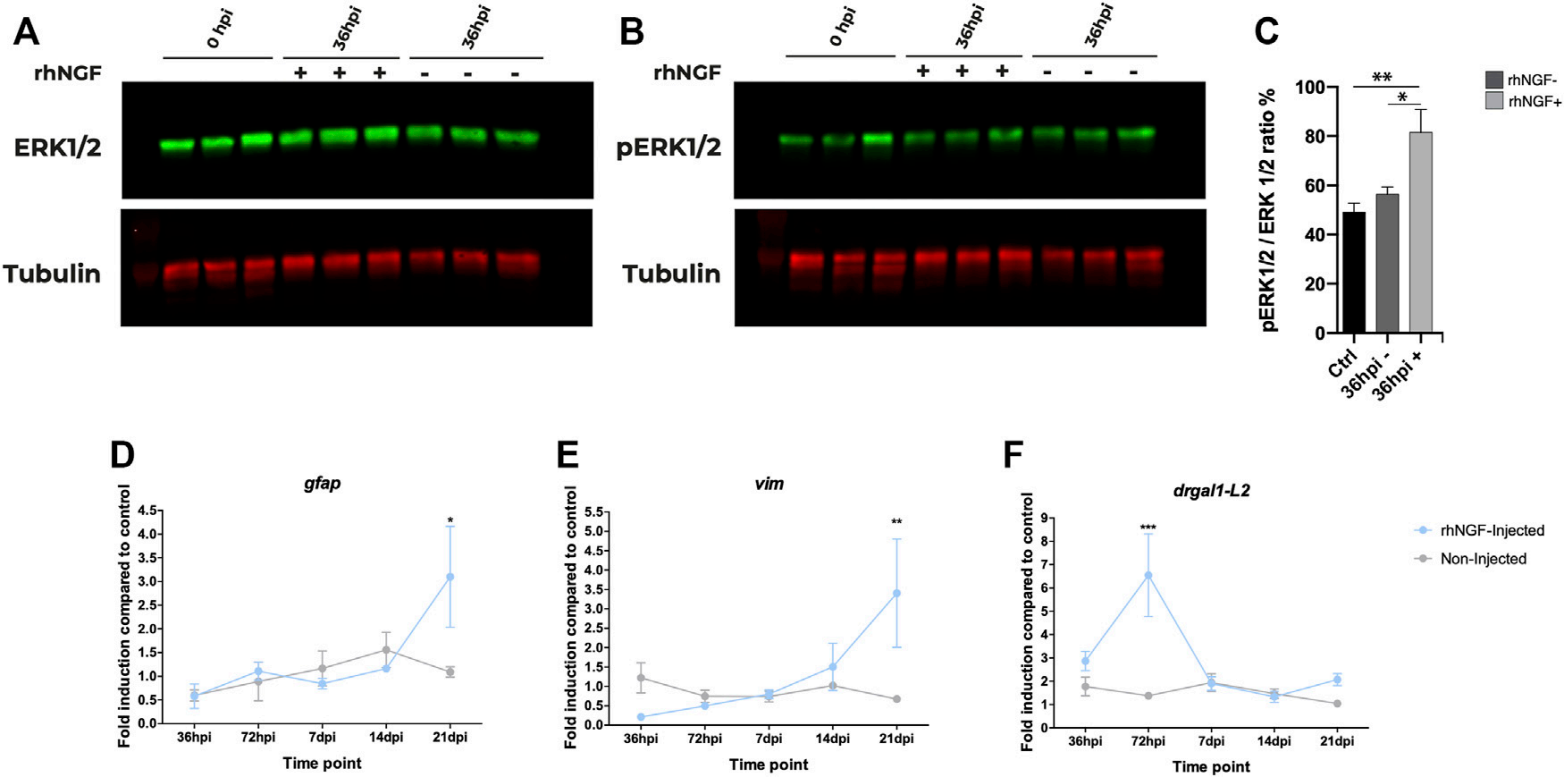
rhNGF-mediated pathway activation and gene expression in adult zebrafish during retinal regeneration. (A, B) Western blot analysis of ERK during retinal regeneration. **(A)** Total ERK protein levels in non-injected and rhNGF-injected adult zebrafish eyes at 0 h post injury (hpi) and at 36 hpi. Ctr-0 hpi (*N*: 15 eyes); 36 hpi rhNGF − (*N*: nine eyes); 36 hpi rhNGF + (*N*: 9 eyes) (3 eyes × time point × condition). **(B)** Phosphorylated ERK protein levels in the same samples analyzed in A. **(C)** Bar plot of pERK/ERK ratio as normalized fluorescence intensity relative to protein levels. Data are shown as means and standard error of the mean (SEM). The statistical analysis test used was one-way ANOVA followed by a Dunnett’s multiple comparisons test; 0 hpi vs. 36 hpi rhNGF +: Adjusted *p*-value: 0.0078; 36 hpi un-injected *N*: nine eyes; 36 hpi rhNGF − vs. 36 hpi rhNGF +: Adjusted *p*-value: 0.0347. **(D–F)**. Expression levels of Müller glia-specific genes *gfap*, *vim*, and *drgal1-L2* during retinal regeneration (*N* three eyes time point condition). Data are shown as mean ± SEM. ***p < 0.001; **p < 0.01; *p < 0.05 (two-way ANOVA test followed by a Sidak’s multiple comparisons test). *gfap*: 21 dpi un-injected vs. 21 dpi rhNGF-injected. Adjusted *p*-value: 0.0134; *vim*: 21 dpi un-injected vs. 21 dpi rhNGF-injected: Adjusted *p*-value: 0.0052. *drgaI*: 72 hpi un-injected vs. 72 hpi rhNGF-injected: Adjusted *p*-value: <0.001. rhNGF: recombinant human nerve growth factor.

In order to determine whether IV injection of rhNGF led to the activation of NGF canonical pathway, we analyzed the changes in ERK protein expression levels, a known NGF downstream effector, after light-induced retinal degeneration. The phosphorylation of these proteins indicates activation of the pathway; therefore, the ratio between phosphorylated ERK and total protein was evaluated. A significant increase in phosphorylated ERK levels was observed 36 h after the IV injection in eyes treated with rhNGF compared to non-injected contralateral eyes and to eyes extracted immediately after 60 h of light injury (Figures 5A–C), suggesting that rhNGF administration is able to induce activation of the canonical pathway.

We next sought to evaluate the effect of rhNGF injection on regenerative mechanisms investigating the potential role of rhNGF on MG by analyzing the expression of genes specific of this cell population, *gfap* (glial fibrillary acidic protein) and *vim* (vimentin) (Figure 5D,E) (Ranski et al., 2018). In order to detect potential changes in expression from early activation to later regeneration phases, for the expression analysis, we selected a range of time spanning from 36 h post injury (hpi) to 21 dpi. Since the peak of photoreceptor apoptosis has been shown to occur at 24 hpi and is followed by MG cell-cycle re-entry at approximately 30 hpi (Vihtelic and Hyde 2000), changes in gene expression were evaluated at two additional time points (36 and 72 hpi) not included in the ONL regeneration analysis, while 28 dpi was identified as a *plateau* stage for regeneration analysis and therefore was considered not informative. No differences in expression levels were detected at the analyzed time points, with the exception of the last one. At 21 dpi, in fact, transcriptional levels of both *gfap* and *vim* increased threefold in rhNGF-injected eyes compared to the control eyes.

To investigate the mechanisms underlying the late effect of NGF on MG, we also analyzed also the expression of *drgal1-L2* (galectin 1–like 2), a secreted factor that is expressed by microglia and regenerative MG and their lineage starting approximately 24 hpi, which is required for rod photoreceptor regeneration (Marc et al., 2003) and thus represents a molecular signature of regenerating zebrafish retina. At 36 hpi, an upregulation of *drgal1-L2* transcription was observed in both injected and untreated eyes compared to the basal expression level (2 folds change); however, at 72 hpi, a drastic threefold increase was detected in rhNGF-injected retinal tissues compared to the untreated ones.

Overall, these results suggest that activation of NGF pathway by injection of exogenous growth factor might enhance the regenerative potential of the tissue, and this effect is possibly mediated by a rhNGF-induced proliferation of MG cell populations in the early post-injury time points, which would result in an increased size in the pool of MG in later time points.

## Discussion

Retinal degeneration and damage progressively lead to low vision and blindness, and no radical treatment is currently available (Jin and Takahashi 2012). Here, we evaluated the potential retinal regenerative effects of intravitreal injection of rhNGF in a model of light-induced retinal degeneration in adult zebrafish.

NGF and its receptors, TrkA and p75, are widely expressed in mammal central visual pathway (lateral geniculate nucleus and visual cortex), as well as in the optic nerve and retina (Chakrabarti et al., 1990). In the neural retina of adult rodents, NGF is mainly expressed by retinal ganglion cells (RGCs) and glial cells, like Müller cells and microglia, as well as by photoreceptors and in photoreceptor outer segments (Sun et al., 2008). The presence of TrkA and p75 in photoreceptors, RGCs, and Müller cells suggests that these cells can respond to NGF signaling, which might modulate their survival or death (Garcia et al., 2017). In line with this hypothesis, it has been shown that exogenous NGF protects retinal cells from degeneration and apoptosis in experimental retinal detachment (Sun et al., 2007) and in models of RP (Rocco et al., 2015; Sacchetti et al., 2017). In zebrafish, the expression of NGF receptors (ntrk1 and ngfrb) had been investigated and reported only during the early development of the nervous system (Nittoli et al., 2018; Hahn et al., 2020), while no data are available regarding the expression of these receptors in the adult fish. In this context, our result demonstrated the presence of *ntrk1* and *ngfrb*, orthologues of TrkA and p75 in different layers in the retina of adult zebrafish and represent the first piece of evidence of the conservation of NGF retinal expression pattern between adult mammals and fish, suggesting that the potential therapeutic effects of rhNGF can be assessed in a zebrafish model of retinal degeneration.

During the last years, zebrafish have been extensively studied to understand molecular mechanisms underlying retinal regeneration, which, in this organism, lasts throughout the entire lifespan (Wan and Goldman 2016). Models of retinal disease have been described in zebrafish and mouse based on bright or UV light irradiation that leads to photoreceptor cell apoptosis and degeneration of the sensory retina (Belmonte et al., 2006; Saito et al., 2016). However, the reported experimental procedures vary among research groups and present different degrees of phenotype severity, as a standardized protocol has not been established yet. Some models involve the exposure of the animals to a constant bright light of a density of ∼8,000 lux (Vihtelic and Hyde 2000; Thomas et al., 2012) or even less, i.e., 2,800 lux (Thummel et al., 2008a), for a prolonged time (up to 4 days) to obtain significant reduction of outer nuclear layer (ONL) thickness and increase of apoptotic cells. Other studies, however, have reported the establishment of a light-induced retinal degeneration model in adult pigmented zebrafish using a greater light density (∼16–20,000 lux). Under these conditions, Rajaram et al. (2014) described severe damage of ONL at 51 hpi, while in another study (Saito et al., 2016), the effect on fish retinae could be achieved even by a lower exposure time (up to 48 h). Starting from these previous reports, we irradiated fish with a constant light intensity of 18,000–20,000 lumens and carefully set up our model checking for retinal damage at 48 and 60 h, finally choosing this latter time point based on ONL thickness and cell number. In the present study, we thus defined a robust experimental procedure for light-induced retinal degeneration based on 60 h of constant bright light irradiation. We have shown that such exposure causes photoreceptor apoptosis, which leads to a striking reduction of ONL thickness and cell number, the readouts used to assess retinal tissue damage. In this model, IV administration of rhNGF speeded up the amelioration of damaged retinal tissue in treated animals compared to untreated siblings, which was especially evident and significant in the late stages of regeneration. In fact, injected and control retinae showed a similar phenotype during the first phases of regeneration (7 dpi), while a significant improvement in ONL thickness and cell number was observed at later (14 and 21 dpi) time points. These results suggest that rhNGF might activate underlying molecular mechanisms in the early regeneration processes, such as MG dedifferentiation and proliferation, and this would result in faster recovery from damage at late stages of regeneration, until reaching the plateau in the final phases.

In zebrafish, the primary source of regeneration is the MG, which can generate and replace all types of retinal neurons after injury (Ramachandran et al., 2010). Thus, the improvement that we observed in ONL thickness and cell number after the treatment with NGF could be the result of two processes: on one side, NGF could have stimulated MG division, thus leading to the repopulation of retinal tissue through the differentiation of different classes of retinal neurons; on the other hand, NGF-induced proliferation could have led to an expansion of the MG pool at later time points, when the regeneration process was completed. The high expression of proliferating cell nuclear antigen (PCNA) that we observed in NGF-treated retinae and data from a seminal work demonstrating the mitogenic effect of NGF in culture MG and the expression of NGF receptors in this cell population (Ikeda and Puro 1994) might validate the second scenario supporting a direct proliferating effect of NGF on the MG.

Analysis of the downstream effectors of NGF in treated zebrafish revealed a significant upregulation of the ERK1/2 pathway at 36 hpi in rhNGF-injected eyes compared to controls, indicating that injection of rhNGF activates the pathway at early time points inducing cell proliferation, which accounts for the enhanced retinal regeneration documented by later ONL thickness improvement and increment of MG-specific gene expression, *gfap* and *vim*. In line with this, the injection of rhNGF also drastically increased the levels of Drgal1-L2, a secreted factor that plays a key role in retinal development and photoreceptor regeneration. This upregulation of regeneration-specific gene programs at 72 hpi, in association with the faster recovery of the photoreceptor cell layer observed by cytological analysis, indicates that rhNGF treatment boosts the regenerative potential of zebrafish retinal tissue upon injury.

Although the use of zebrafish as a model to test the effects of therapeutic or neuroprotective compounds has been scarce until now (Cunvong et al., 2013; Saito et al., 2016), our results on NGF receptors’ expression and pathway activation in adult zebrafish strongly sustain the potential successful exploitation of zebrafish as a powerful alternative model for testing the efficacy of novel IV injection-based treatments for retinal diseases. Moreover, the regenerative effects that we observed in zebrafish retinae upon NGF treatment have an important translational impact. An increasing body of evidence in fact indicates that MG retains multipotency and can be reprogrammed in neurons to restore cellular function in damaged retina stem cells also in adult mammals (Yao et al., 2018), and thus similar regenerative effects can be expected after the treatment with rhNGF also in mammal MG, potentially leading to retinal regeneration after injury or degeneration.

In light of our data and considerations, our study opens the way to the use of zebrafish as a useful model to test new compounds potentially able to stimulate the regeneration properties of the retina and paves the ground to further studies aimed at evaluating in particular the effects of IV-injected NGF also in mammals, in order to expedite the development of novel and promising alternative therapeutic approaches for ophthalmological indications based on rhNGF administration.

## Materials and Methods

### *In vivo* Experiments

Zebrafish (*Danio rerio*) were maintained at 24°C on a 12-h light/12-h dark cycle. Collected embryos were cultured in fish water containing 0.01% methylene blue to prevent fungal growth. Fish were grown in the fish facility of ZeClinics SL. Light-induced retinal damage protocol was adapted and optimized from previous reports (Belmonte et al., 2006; Saito et al., 2016). Adult fish anesthetized with 100 mg/L of MS-222 (Millipore Sigma, St. Louis, MO) were positioned on one side and the outer cornea was removed. Next, a small hole was opened close to the lens and 5 µg of rhNGF (Dompé farmaceutici s.p.a) was injected intravitreally, after 60 h of light irradiation (60 h LID). All procedures performed were in accordance with Spanish and European Union animal welfare guidelines and approved by the Animal Testing Ethics Committee (CEEA; n:20–005-ISA).

### Immunohistochemistry and Imaging

Following euthanasia with 300 mg/L of MS-222 at 0, 7, 14, 21, and 28 dpi, zebrafish eyes were removed, fixed in 4% paraformaldehyde/1 × phosphate buffered saline (PBS; pH 7.4) overnight at 4°C and cryoprotected overnight (O/N) in 30% sucrose/0.02% sodium azide/PBS before embedding in O.C.T. compound (Sakura Finetech, Torrance, CA). Embedded samples were then frozen on dry ice, and 14-μm sections were mounted on Fisherbrand Superfrost Plus slides (Fisher Scientific, Waltham, MA). Cryosections were washed three times in 1 × PBS/0.1% Tween-20 (PBS-T) solution and incubated for 1 h at room temperature (RT) in 10% Normal Goat Serum (ThermoFisher, Carlsbad, CA) in PBS-T blocking solution. Sections were stained O/N at 4°C with mouse Zpr1 antibody (1:250) (AbCam, Cambridge, United Kingdom), a monoclonal antibody that recognizes zebrafish cone arrestin 3a, which is expressed specifically by both red and green cones from the synaptic pedicle of the apex of the inner segment. Goat anti-mouse IgG AF594 antibody (1:200) (ThermoFisher, Carlsbad, CA) and DAPI (4′,6-diamidino-2-phenylindole) DNA fluorescent stain (1:500) (Sigma) in blocking solution were added for 2 h at RT. Sections washed in PBS-T were mounted with Vectashield mounting media (Vector Laboratories, Burlingame, CA). Slides were left at RT for 1 h before imaging with a Leica SP5 confocal microscope. Images were analyzed with ImageJ software 3, 25. Statistical significance was assessed with GraphPad Prism One-way ANOVA followed by Dunnett’s or Sidak’s test.

### Gene Expression Analysis by Real-Time PCR

At 36 hpi, 72 hpi, 7 dpi, 14 dpi, and 21 dpi, three eyes per condition were homogenized in TRIreagent (MilliporeSigma), and total RNA was extracted following the manufacturer’s protocol. RNA concentration was estimated using a NanoDrop Spectrophotometer ND-1000 (NanoDrop Technologies; Wilmington, DE). One hundred nanograms of RNA was retrotranscribed to cDNA by reverse transcriptase (Superscript III RT-Enzyme, ThermoFisher) and stored at −20°C. Gene expression was assessed with a Lightcycler^®^ 480 system (Roche) using the SYBR GREEN method from 1:10 cDNA and β-actin as a housekeeping gene (stability determined by Bestkeeper^©^ software). Relative expression levels were quantified using the ΔΔCt method.

### Riboprobe Synthesis for *In Situ* Hybridization (ISH)

*In vitro* transcription of probes was performed using RNA Labeling Kit (Roche) following the manufacturer’s instructions. cDNAs were amplified by PCR from a custom zebrafish cDNA library obtained by RT-PCR performed on mRNA from 4 months adult zebrafish. SP6 sequence linker was included in reverse primers to directly use synthesized PCR products as templates to amplify the reverse riboprobe for ISH. After ISH, embryos were analyzed on a Leica M165 FC microscope. Images were processed using Adobe Illustrator software.

### *In Situ* Hybridization on Cryosections

Slides were digested with 20 μg/ml proteinase K in pre-warmed 50 mM Tris for 10–20 min at 37°C and thus rinsed 5 × in distilled water. Afterwards, sections were immersed in ice-cold 20% (v/v) acetic acid for 20 s and dehydrated by washing for approximately 1 min per wash in 70% ethanol, 95% ethanol, and 100% ethanol. Hybridization solution (100 µl) was then added to each slide. The slides were incubated for 1 h in a humidified hybridization chamber at 62°C while the probes were diluted in hybridization solution and heated at 95°C for 2 min. We added 100 μl of diluted probe per section and incubated the slides in a humidified chamber at 65°C overnight. Cover the sample with a cover slip to prevent evaporation. Thus, we performed stringency washes to remove non-specific bindings. Thereafter, slides were washed twice in MABT (maleic acid buffer containing Tween 20) for 30 min at room temperature. We proceeded with blocking and revealing according to standard protocol by Abcam (https://www.abcam.com/protocols/ish-in-situ-hybridization-protocol).

### PCNA Detection and TUNEL Detection

The 14-μm sections were mounted on Fisherbrand Superfrost Plus slides. Prior to anti-PCNA immunostaining, an antigen retrieval step has been performed. Slides were incubated with 10 mM NaCitrate 0.05% Tween 20 Buffer pH 6.0 in a steamer for 20 min and then cooled down at room temperature for 30 min. Immunohistochemistry has been performed incubating the slides overnight at 4°C with anti-PCNA primary antibody diluted 1/500 in blocking solution. After three washes with PBST, slides were incubated for 2 h at room temperature with anti-Mouse Alexa Fluor 488 secondary antibody diluted 1/500 in blocking solution and finally washed with PBST. For TUNEL assay, retinal cross sections were fixed with 4% PFA for 15 min at RT and apoptotic cells were labeled with *In Situ* Cell Death Detection Kit and TMR red kit following the manufacturer’s instructions (Roche).

### Image Analysis

Adult fish retinae were cryo-sectioned with 14 μm thickness. For analysis, we used sections of the central part of the dorsal half of the retina. Section orientation was established and maintained throughout the different experiments in order to obtain retinal regions that could be compared in the study. The entire volume of a representative cryosection was imaged with a Leica SP5 confocal microscope (for imaging of samples in Figures 2–4), employing a ×20 objective (1 μm per stack). For quantification of the thickness and cell number parameters, the imaged acquired were processed through ImageJ software. As the thinning of the ONL upon damage makes it difficult to use parameters other than length for the analysis of retinal tissue, we identified a “retinal length unit” in the central part of the dorsal half of the retina, which has been then used as reference for all the experiments. In particular, we established a retinal length of 70 μm as our reference region of interest (ROI) for ONL nuclei counting, which was then used also for comparison with other conditions. A maximum projection of the total number of the stacks was performed for each imaged retina. ONL thickness measurement was performed in Maximum projection images through ImageJ software in the central part of the ROI by using the straight-line tool. ONL cell count measurement was performed in Maximum projection images through ImageJ by using the point selection tool after background subtraction. For PCNA and TUNEL staining, cryosections were analyzed on a Leica DMI6000B microscope system. For quantification, positive cells were counted using the point selection tool after background subtraction. The number of stained cells were then divided by area of retina analyzed. Area was calculated using ImageJ Fiji function “measurement” across the ROI used for the counting. Results were plotted using GraphPad Prism software and statistical significance was assessed using the most appropriate statistical test.

### Western Blot

Three eyes per condition were lysed in 150 ml of RIPA buffer (MilliporeSigma) plus proteinase inhibitors (Roche), homogenized, incubated on ice for 15 min, and finally sonicated for 15 s. Samples were kept on ice for an additional 15 min and centrifuged for 20 min at 4°C. Supernatant was stored at −80°C. Proteins were quantified using Bradford assay (MilliporeSigma). Twenty milligrams of protein per sample was combined with 3 ml of 5 × Laemmli loading buffer and water to a final 15-ml volume, boiled at 98°C for 5 min, and loaded onto a 10% SDS-PAGE gel (Bio-Rad, Hercules, CA). Proteins were transferred to a PVDF membrane, blocked for 1 h at RT, and incubated with antibodies for MAPK (1:500) and pMAPK (1:1,000) (Cell Signaling Technology Danvers, MA) in blocking buffer (Licor, Lincoln, NE) diluted 1:2 with TBS-T (0.2%) O/N at 4°C. Blots were then incubated in secondary antibodies (1:5,000) (Licor) in TBS-T for 1 h at RT. Antibody against tubulin (Sigma) and its corresponding secondary were incubated for 1 h at RT. Blots were developed with Odyssey instrument and analyzed using Image Studio Lite software.

### Primers for ISH

A list of the primers used for ISH is reported in Table 1.

**TABLE 1:** List of the primers used for ISH.

Gene	Protein encoded	Transcript ID	Primer name	Sequence
*ngfra*	*nerve growth factor receptor a*	ENSDART00000125281.3	*ngfra*Fw	GGA​GTG​ATC​AAG​GAG​TGT​GGA​G
*ngfra*	*nerve growth factor receptor a*	ENSDART00000125281.3	*ngfra*Rev	AGA​TGG​GGA​TCA​GGT​TCT​CAT​TTA​G
*ntrk1*	*neurotrophic tyrosine kinase, receptor, type 1*	ENSDART00000027013.5	*ntrk1*Fw1	TAG​ACC​TCG​CCA​ACT​ACA​GAG​A
*ntrk1*	*neurotrophic tyrosine kinase, receptor, type 1*	ENSDART00000027013.5	*ntrk1*Rev1	TCC​GTT​GAT​TGG​TTA​GTG​GGA​G
*ntrk1*	*neurotrophic tyrosine kinase, receptor, type 1*	ENSDART00000027013.5	*ntrk1*Fw2	AAT​GGC​TGT​ACA​ACA​ACA​TGC​C
*ntrk1*	*neurotrophic tyrosine kinase, receptor, type 1*	ENSDART00000027013.5	*ntrk1*Rev2	TTC​ACC​CAG​TTC​CCA​CTT​CAA​A
*ntrk1*	*neurotrophic tyrosine kinase, receptor, type 1*	ENSDART00000027013.5	*ntrk1*Fw3	TTT​GAA​GTG​GGA​ACT​GGG​TGA​A
*ntrk1*	*neurotrophic tyrosine kinase, receptor, type 1*	ENSDART00000027013.5	*ntrk1*Rev3	CCT​TGA​TGA​CCA​ACC​TTT​GCT​G

### Primers for Real-Time PCR

A list of the primers used for real-time PCR is reported in Table 2.

**TABLE 2:** List of the primers used for real-time PCR.

Gene	Primer sequence
*ntrk1_fw*	CTA​TGC​CTA​CAC​TGA​ACT​CAT​C
*ntrk1_rv*	TGC​CCA​GTT​TGT​TCT​TCA​C
*ngfrb_fw*	TCT​GTC​AAG​ATT​TCG​ATG​CTC​CT
*ngfrb_rv*	GCT​CTC​CGT​AGG​ATT​GTC​CG
*drgal1-L2_fw*	TGT​GCA​ATT​CAT​TCC​AGA​GC
*drgal1-L2_rv*	AAC​CCC​TTG​GAT​CCT​GAC​TT
*vim_fw*	TAA GCC TGC GAG AGT CCA TGA
*vim_rv*	TCG TTT TGG GTG GAC TCG TT
*gfap_fw*	GCA GAC AGG TGG ATG GAC TCA
*gfap_rv*	GGC CAA GTT GTC TCT CTC GAT C

## Data Availability

The original contributions presented in the study are included in the article/Supplementary Materials, further inquiries can be directed to the corresponding authors.
