# Supplementary material for: Intravitreal Administration of rhNGF Enhances Regenerative Processes in a Zebrafish Model of Retinal Degeneration
Source: Front Pharmacol. 2022 Mar 7;13:822359. doi: 10.3389/fphar.2022.822359 (PMC8940169; doi:10.3389/fphar.2022.822359)
Supplement: Supplementary file 1 [file DataSheet1.docx]

Supplementary Material

# Supplementary Figures


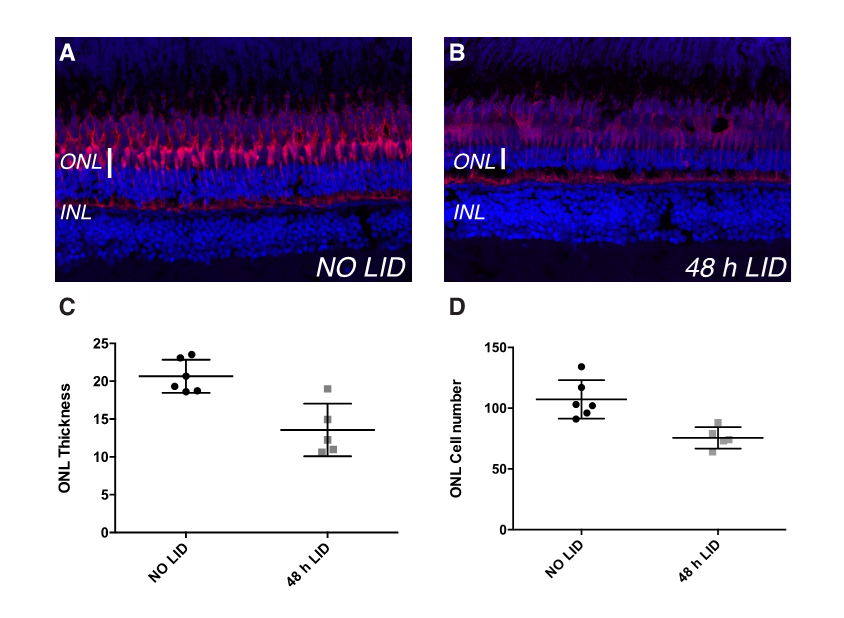


**Supplementary Figure 1.** rhNGF Setup of a light-induced retinal degeneration paradigm. A, B. Representative images of retinal cryosection of adult zebrafish eyes immunostained for Zrp1 (red) and stained with DAPI (blue). A. Retinal cryosection of adult zebrafish eye not exposed to constant light (Ctr). B. Retinal cryosection of adult zebrafish eye exposed to 48 hours of constant light (Light Induced Degeneration- LID). C. Quantification of ONL thickness (number of retinae analyzed = 5-6). D. Quantification of ONL cell number (number of retinae analyzed = 5-6)


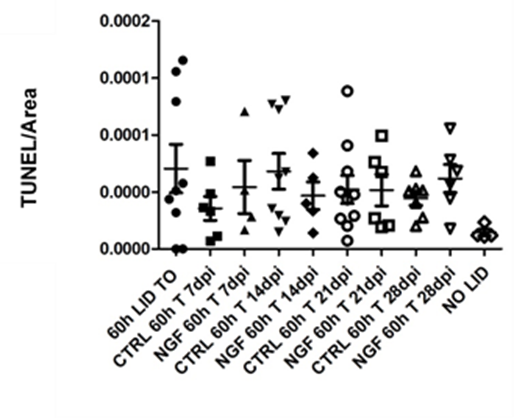


**Supplementary Figure 2.** Quantification of TUNEL positive cells upon Light Induced Degeneration (LID). Ratio of TUNEL positive cells over analyzed area is shown (number of retinae analyzed = 10 per condition). Data are shown as means ± SD (n= 4-10).
